# Retrieving spin textures on curved magnetic thin films with full-field soft X-ray microscopies

**DOI:** 10.1038/ncomms8612

**Published:** 2015-07-03

**Authors:** Robert Streubel, Florian Kronast, Peter Fischer, Dula Parkinson, Oliver G. Schmidt, Denys Makarov

**Affiliations:** 1 Institute for Integrative Nanosciences, IFW Dresden, 01069 Dresden, Germany; 2 Helmholtz-Zentrum Berlin für Materialien und Energie GmbH, Berlin 12489, Germany; 3 Center for X-ray Optics, Lawrence Berkeley National Laboratory, Berkeley, California 94720, USA; 4 Department of Physics, UC Santa Cruz, Santa Cruz, California 95064, USA; 5 Advanced Light Source, Lawrence Berkeley National Laboratory, Berkeley, California 94720, USA; 6 Material Systems for Nanoelectronics, TU Chemnitz, Chemnitz 09107, Germany

## Abstract

X-ray tomography is a well-established technique to characterize 3D structures in material sciences and biology; its magnetic analogue—magnetic X-ray tomography—is yet to be developed. Here we demonstrate the visualization and reconstruction of magnetic domain structures in a 3D curved magnetic thin films with tubular shape by means of full-field soft X-ray microscopies. The 3D arrangement of the magnetization is retrieved from a set of 2D projections by analysing the evolution of the magnetic contrast with varying projection angle. Using reconstruction algorithms to analyse the angular evolution of 2D projections provides quantitative information about domain patterns and magnetic coupling phenomena between windings of azimuthally and radially magnetized tubular objects. The present approach represents a first milestone towards visualizing magnetization textures of 3D curved thin films with virtually arbitrary shape.

## Abstract (web summary)

The determination of the three-dimensional magnetic domain structures of nanostructures is an ongoing challenge. Here, Streubel *et al.* use two-dimensional X-ray microscopy to reconstruct three-dimensional domain structures in ferromagnetic thin-film cylinders.

## Graphical abstract

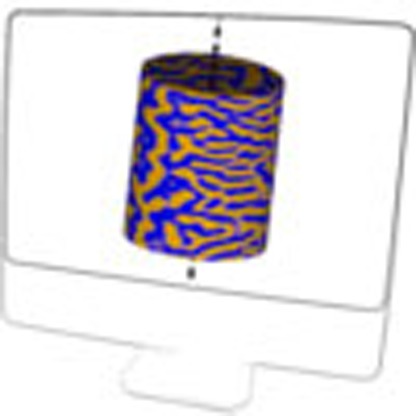

High penetrability and short wavelengths of electron, neutron and X-rays allow for developing tomographic imaging techniques^1^, which reveals internal structures in complex organic^2^,^3^,^4^ and inorganic^5^,^6^,^7^,^8^,^9^,^10^,^11^ objects. In particular, conditions during formation of minerals^5^ and ceramics^6^, functioning and interplay of biological cells at the subcellular level^2^,^3^ had been identified. Magnetic macro- as well as nanosized objects have been investigated using magnetic neutron tomography^7^,^8^ and electron-based techniques, including electron holography^4^,^10^,^12^,^13^,^14^ and vector field electron tomography^9^,^11^, respectively. Those techniques rely on the detection of the phase shift of the probing radiation originating from the interaction with magnetic fields. In particular, neutron tomography is sensitive to magnetic domain walls^15^, whose three-dimensional (3D) spatial distribution can be derived with a spatial resolution of about 100 μm from a set of projections via direct iterative reconstruction without applying the Fourier slice theorem^16^. First successful attempts to access magnetic properties in 3D space with nanometre resolution have been accomplished in uniformly magnetized nanoparticles^12^,^13^, nanorods/nanospirals^10^,^14^ and magnetotactic bacteria^4^,^13^ with spatial expansions <100 nm by phase shift reconstruction with electron holography^17^,^18^ or Lorentz electron microscopy and employing the transport-of-intensity equation^19^,^20^. The loss of local information about the phase shift can be avoided by acquiring projections at different angles while rotating the sample around at least two axes. The vector field electron tomography^21^,^22^ provides means to fully reconstruct 3D magnetic vector fields as demonstrated recently for magnetic vortex cores in soft-magnetic disks^9^,^11^.

In addition to traditional 3D magnetic materials, that is, bulks and nanostructures, a new class of objects has recently emerged. Magnetic thin films with thicknesses in the 50-nm range extending over tens of micrometres, which are shaped into 3D configurations, resemble novel magnetic 3D structures. These mesoscale systems possess striking novel properties originating from curvature-driven effects, such as magnetochiral effects^23^,^24^,^25^,^26^,^27^,^28^,^29^ and topologically induced magnetization patterns^27^,^30^ due to a Dzyaloshinskii-like interaction^31^. Moreover, tubular shapes with circulating^32^,^33^ and radial magnetization^34^,^35^ are not only appealing from a fundamental point of view, but also highly relevant for applications, such as in medicine as an isotropic highly sensitive magnetoimpedance-based sensor for encephalography^36^,^37^ or as novel concepts in data storage^38^,^39^. Typically, those structures possess complex multidomain states that determine their magnetic response. As characteristic lengthscales of thin magnetic films, such as domain size and domain wall width, are in the tens of nanometre regime, a nanometre spatial resolution is required to image details of the magnetic domain pattern even if the curvature radii of these 3D surfaces is in the micrometer range. The weak interaction of neutrons with matter and the limited spatial resolution prohibits neutron tomography from investigating thin film magnetic 3D structures. On the other hand, electron holography is challenging given the lateral dimensions and thicknesses of the sample as well as radii of curvature. Hence, a technique that provides direct access to the magnetization of mesoscopic 3D-shaped thin films with nanometre spatial resolution and element specificity is highly demanded. Magnetic soft X-ray tomography (MXT) has the potential to become a powerful tool to address the unique magnetic responses of an entirely new class of 3D magnetic objects with arbitrary shape and magnetization.

Here we put forth the foundation of imaging magnetic 3D thin films with X-ray microscopies and demonstrate its capabilities by reconstructing complex 3D spin textures of hollow cylinders. Using full-field soft X-ray microscopies, such as X-ray photoemission electron microscopy (XPEEM) operated in transmission mode (T-XPEEM) and magnetic transmission soft X-ray microscopy (MTXM), harnessing X-ray magnetic circular dichroism (XMCD) as element-specific magnetic contrast mechanism^40^,^41^,^42^,^43^, we can study magnetic thin films of up to 200 nm in thickness, which is limited by the penetration depth of soft X-rays. Applying these techniques, we are able to record an angular scan (0–180°) of projections of the 3D magnetization distribution onto a two-dimensional (2D) plane. While simple spin textures like circulating magnetization textures can be retrieved from the experimental data by correlating them with XMCD contrast simulations in the projected patterns, more complex domain patterns require proper tomographic reconstruction algorithms. We report the mathematical algorithm to reconstruct 3D magnetic patterns of radially magnetized tubular objects. Thus, fundamental insight into the morphology of the domain pattern (shape and size of domains) and magnetic coupling phenomena in 3D curved surfaces is obtained, revealing an angular dependence of both domain size and shape that can be understood and correlated to the Kondorsky mechanism of magnetization reversal^41^,^44^. A quantitative analysis of these domain patterns addresses the transition from isotropic to azimuthally aligned magnetic domains of the remanent state. The present work is a first milestone in visualizing the magnetization textures of 3D curved thin films with tubular shape utilizing the XMCD effect with full-field soft X-ray microscopies. It paves the way for future advancements of the reconstruction algorithms towards investigating curvature-driven effects in 3D objects with virtually arbitrary shape.

## Results

### Concept of MXT

**Figure 1:**
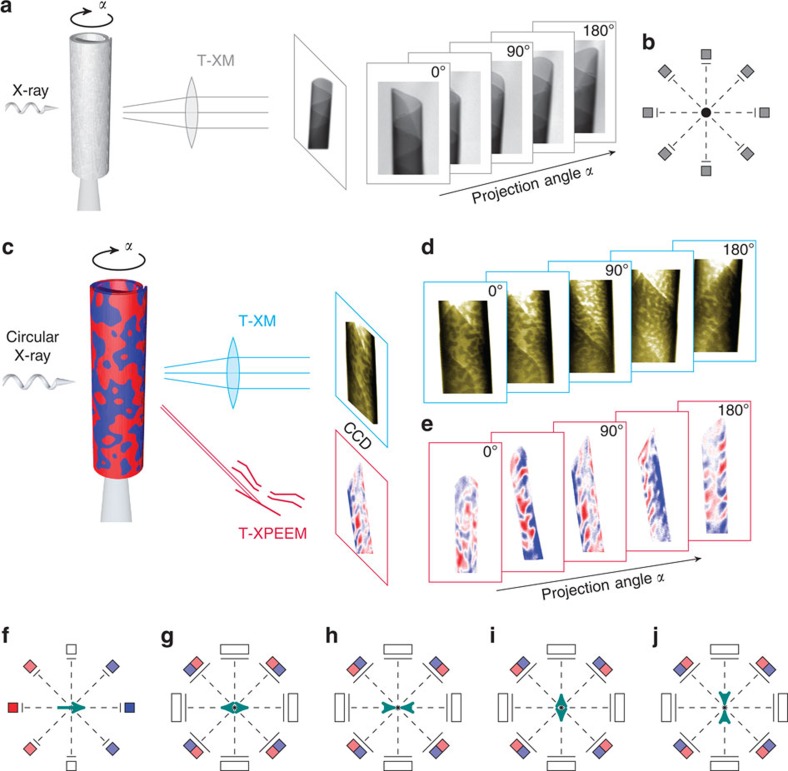
Comparison of the conceptual difference between conventional scalar tomography and MXT. (**a**) Light is attenuated when penetrating an object, for example, rolled-up tubular magnetic nanomembrane, according to its local atomic mass distribution. (**b**) The spatial distribution in 3D space is obtained by applying tomographic reconstruction algorithms relying on the angle invariance of the scalar properties. (**c**) The magnetization component along the beam propagation direction can be visualized utilizing XMCD with soft X-ray microscopies. (**d**,**e**) exemplarily show the XMCD contrast patterns of radially and in-plane magnetized tubular architectures recorded with a TXM and a T-XPEEM, respectively. (**f**) The XMCD signal shows an angular dependence that even reverses its sign for space-inverted X-ray propagation (red to blue). The corresponding underdetermined system of projections leads to an ambiguity of possible reconstruction when considering arrangements of two or more macrospins (**g**–**j**) that may only be released by locating the magnetic material, determining the preferential magnetization orientation and analysing their evolution with varying projection angle. Determining all magnetization vector components requires to record projections around several rotation axes. The present case of tubular surfaces with either radial or in-plane magnetization allows for retrieving the magnetization textures from one set of projections taken around a rotation axis that coincides with the symmetry axis.

Compared with X-ray tomography (Fig. 1a), which generates a map of the X-ray absorption in the 2D projection images due to the interaction with charges (Fig. 1b), that is, a scalar density distribution, the challenge with MXT is to retrieve the vector density distribution of the magnetization (Fig. 1c–e). The term tomography used in this manuscript refers to the possibility to reconstruct the 3D spatial distribution from a set of 2D projections. The basics of and emergent challenges with that method when applied to XMCD-based microscopy can be illustrated in the following example. The XMCD signal contains information about the projection of the magnetization vector onto the X-ray beam propagation direction, which leads to an angle-dependent signal contribution of the same magnetization vector (Fig. 1f) and a possible XMCD contrast compensation. Red and blue contrast refer to magnetization vectors with the same amplitude but opposite direction. The absence of a net XMCD contrast is indicated by white. In case of a non-uniform magnetization distribution, the vector property-induced non-additivity of the XMCD signal causes a complex ambiguous projection of the 3D magnetization, as exemplarily shown for radially and in-plane magnetized tubular surfaces in Fig. 1d,e, respectively. Let us consider the simplest case of two identical macrospins aligned antiparallel to each other, which occurred, for example, at the front and back side of a radially magnetized thin tube (Fig. 1g–j). The projections along and perpendicular to the magnetization vector become zero due to net moment compensation and zero projection onto the X-ray propagation direction, respectively. However, an illumination at, for example, ∼45° with respect to the macrospin direction results in a non-zero signal that constitutes of laterally separated reddish and bluish contributions with an angle-dependent colour sequence (red–blue or blue–red). Accordingly, two macrospins pointing away from or towards each other can be disentangled. The same projections are also obtained when considering the reversed by 90° rotated case (compare Fig. 1g–j). Note that the angle dependence of these pairs in the range from, for example, 0° to 45° is different and could therefore be used for discrimination. Analysing the contrast evolution with varying projection angle constitutes the idea of MXT. Note that a complete and consistent identification of the magnetization texture requires to record the XMCD contrast around several projection axes and to derive the contrast change, which refers to the derivative of the magnetization vector with respect to the rotation direction. In contrast to vector field electron tomography, it is generally not sufficient to consider only two projection axes when dealing with non-uniform states due to a possible XMCD contrast compensation and locally varying saturation magnetization.

**Figure 2:**
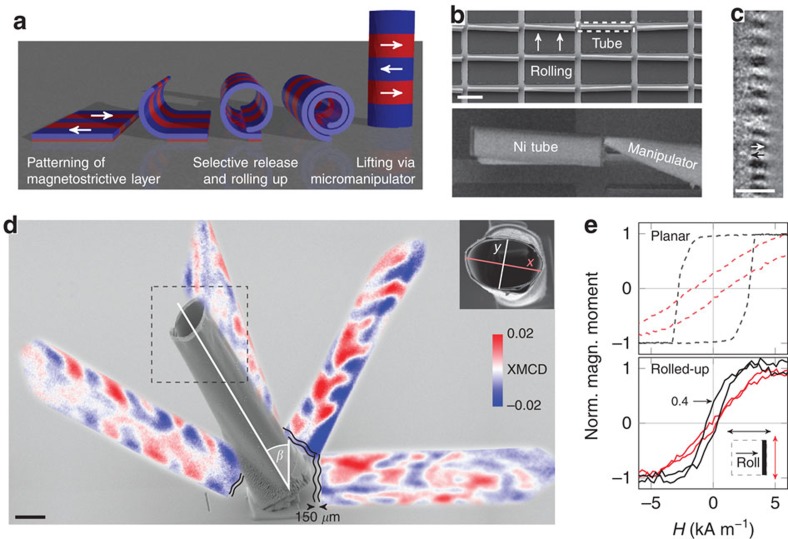
Fabrication and characterization of circulating spin textures. (**a**) Preparation of azimuthally magnetized tubular architectures via selective rolling up of a prepatterned strained nanomembrane with transverse magnetic easy axis (indicated by red and blue, and arrows). (**b**) Electron micrographs of rolled-up tubes and manipulation. Scale bar, 50 μm. (**c**) Transverse magnetization component along a 2-μm wide stripe at the very top of the tube visualized by longitudinal Kerr microscopy. Scale bar, 5 μm. (**d**) Electron micrograph of a vertically fixed Ni tube with circulating magnetization overlaid with XMCD shadow contrast patterns recored by T-XPEEM at various projection angles. The X-ray beam penetrates the top end of the standing tube (dashed rectangle) and projects the magnetization onto the substrate 150 μm away from the tube location (indicated by wiggly lines). The lengths of major and minor axis are *x*=(8.4±0.2) μm and *y*=(5.5±0.2) μm, respectively. Scale bar, 5 μm. (**e**) Magnetic hysteresis loops of planar (dashed) and rolled-up (solid) nanomembranes shown for longitudinal and transverse sensitivity suggest circulating (azimuthal or helical) magnetization within the rolled-up nanomembrane.

**Figure 3:**
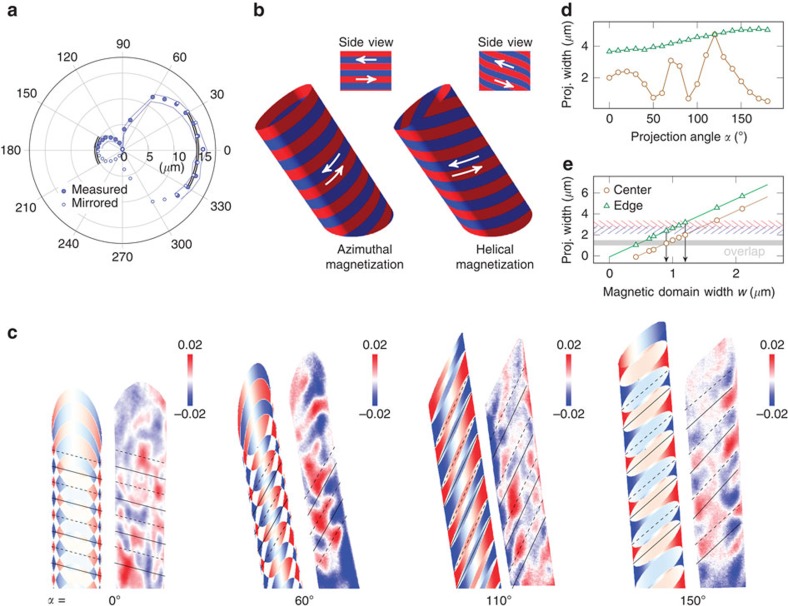
Correlation between simulated and experimentally observed XMCD contrast. (**a**) Angle dependence of the projected ellipse axis along the beam when illuminating at 74° with respect to the surface normal in XPEEM to precisely determine the tube orientation. For 0° and 180°, the tube is tilted towards and away from the beam, respectively. The double lines indicate the analytically calculated periphery. (**b**) Modelled magnetization textures within the rolled-up nickel nanomembrane approximated as hollow tube. Tube geometry is taken from experiment; domain width is *w*=1 μm. (**c**) Correlation of experimental with simulated XMCD contrast of alternating domain widths (0.9 and 1.2 μm (11% and 15% the major axis)) at different projection angles. The projections reveal distinct and complex patterns that can only be assigned by simulation correlation to the corresponding magnetization textures. Solid lines serve as guide to the eye. (**d**) Angle dependence of the projected width shown for 1.7-μm wide magnetic domains. (**e**) Linear dependence of the projected width on the magnetic domain width of an azimuthal magnetization texture at the center line and the edge for *α*=110°. Experimental data (indicated shaded areas) suggests that the domain width is periodically alternating.

**Figure 4:**
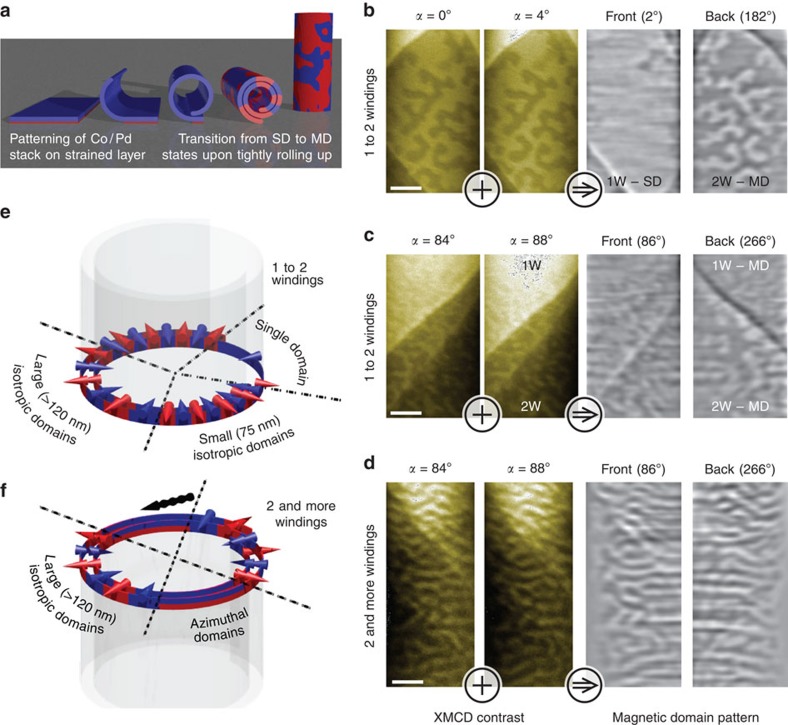
Fabrication and characterization of radially magnetized tubes. (**a**) Preparation of radially magnetized tubes via rolling up out-of-plane magnetized multilayer stacks, for example, Co/Pd multilayers. The normal magnetization component is indicated by red and blue. The initial single domain (SD) state transforms into a multidomain (MD) state on tightly rolling up. (**b**,**d**) show the remanent states of an out-of-plane magnetized rolled-up nanomembrane with 2 μm diameter (field initially applied along 180°) visualized in MTXM. Dark and bright contrast refer to strong and weak absorption, and a magnetization pointing outside and inside the tube, respectively. Regions with one and two windings are indicated by 1 and 2 W, respectively. The magnetization is reconstructed by analysing the evolution of magnetic contrast with varying projection angle *α*, for example, between *α* and *α*+4°. This operation is illustrated by encircled plus and arrow symbols. Scale bars, 500 nm. (**e**,**f**) Schematic illustration of the magnetic domain patterns in tightly wound rolled-up nanomembranes. Large and small domains form along and perpendicularly to the field direction (indicated by arrow), respectively. A multidomain state wrapping around the whole tube as occurring in regions with multiple windings exhibits azimuthally aligned domains.

In extended samples with a rotation axis not coinciding with the local direction of the magnetization vector, the X-ray beam interacts at each projection angle with another set of macrospins. This ambiguity requires to derive valid restriction conditions, such as the spatial confinement of the magnetization vector field by locating the magnetic material using conventional scalar X-ray tomography (Fig. 1a) and the preferential magnetization orientation obtained, for example, from the integral magnetic characterization (Fig. 2). Analysing the evolution of the magnetic signal as a function of the projection angle and taking into account the restriction conditions allows for retrieving the magnetization textures (Figs 3 and 4).

To demonstrate the capability of MXT, we retrieve the magnetic domain patterns in hollow tubes with well-defined magnetization orientations, for example, radial or in-plane. These systems represent 2D or one-dimensional vector fields defined on a hollow cylinder that can be determined from one set of projections recorded around a single rotation axis coinciding with the symmetry axis of the tube. Such structures can be fabricated by different synthesis approaches. Tubes with diameters in the nanometre range can be prepared via electrochemical deposition into porous alumina templates^45^,^46^,^47^,^48^. These objects possess, due to their small dimensions, rather simple domain patterns, for example, longitudinal or azimuthal magnetization, and can easily be studied using electron holography^10^,^14^ or magnetometry^47^,^48^. More complex domain patterns appear in tubular structures with larger diameters in the range of some micrometres, which can be fabricated using strain engineering^49^,^50^. These tubular architectures are particularly appealing as magnetoimpedance sensors for magnetoencephalography^36^,^37^ or as compact giant magnetoresistance sensors for in-flow cytometry^51^,^52^. 2D magnetic nanomembranes with either in- or out-of-plane anisotropy rolled up into tightly wound tubular systems offer well-defined magnetic domain patterns including homogeneous^53^,^54^,^55^ and multidomain states^32^,^33^ or even radial spin textures^34^,^35^. State-of-the-art characterization of their physical properties relies on integral measurement techniques, such as ferromagnetic resonance spectroscopy^54^,^55^, magnetoresistance^33^,^46^,^51^,^56^ and magnetometry measurements^47^,^48^, and on the analysis of 2D projections of magnetization patterns recorded using Kerr^32^,^33^ and X-ray^43^ microscopy. Although these approaches provide information on the magnetic properties, they cannot be applied to reconstruct the 3D magnetic domain pattern. A proper identification can only be accomplished by tomographic imaging.

### Circulating magnetization patterns

As a first example, we analyse the XMCD contrast of simple magnetization textures, such as azimuthal domains with in-plane magnetization, by correlating them to XMCD contrast simulations. This approach is similar to that of electron holography on tubular/helical structures^10^,^14^. The present magnetic tubular architectures with a defined magnetization are prepared via strain engineering^49^,^50^. In particular, lithographically prepatterned strained magnetic nanomembranes are released from the substrate by selectively etching the sacrificial buffer layer and rolled up into tubular architectures by reducing the internal strain gradient (Fig. 2a). The tubular architectures with a diameter of about 7 μm and 2.5 windings (Fig. 2b) and circulating magnetization (Fig. 2c) are picked up and lifted via a micromanipulator inside a cross-beam workstation and fixed onto a Pt-coated Si wafer by ion beam activated Pt deposition (Fig. 2d). The tube exhibits an elliptical cross-section with a major and minor axis of *x*=(8.4±0.2) μm and *y*=(5.5±0.2) μm, respectively, and is tilted by *β*=(31±1)° with respect to the surface normal. Using nickel as both strained and functional magnetic layer provides a negative magnetostriction constant and therefore a transverse magnetization before rolling up (Fig. 2e). After rolling up, a low remanence magnetization of 0.4 times the saturation magnetization was found by longitudinal magneto-optical Kerr effect magnetometry. This is indicative for a circulating magnetization with azimuthal or helical alignment, which could have been verified by Kerr microscopy (Fig. 2c). As the limited depth of focus of Kerr microscopy allows only for visualizing a very narrow stripe along curved tubes (Fig. 2c), different types of circulating magnetization cannot be distinguished. Using T-XPEEM, this limitation can be overcome by projecting the 3D magnetization onto the planar substrate^43^.

The spatial orientation of the tube with respect to the X-ray beam propagation direction inside the XPEEM is determined by analysing the angle-dependent projection of the elliptical cross-section of the tube onto the planar substrate (Fig. 3a). Tube orientations with a tilt towards and away from the incidence direction of the X-ray beam are referred to as 0° and 180°, respectively. The magnetization of the standing tube is visualized by imaging the secondary photoelectrons generated in the substrate after penetrating the tube by circularly polarized photons with an energy corresponding to the Ni *L*_3_ absorption edge. The number of transmitted photons depends on the local magnetization due to the XMCD effect; thus, the projected magnetic pattern reflects the local magnetization in the tube. The long focal point of the X-ray beam of 32 cm provides equal foci for front and back side of the tube and ensures a parallel projection of the magnetization texture. Virtually any 3D magnetic thin film of arbitrary shape on- or off-chip can be investigated in this way. Although the magnetic domains do not change during illumination, their projections at various angles reveal distinct features in both domain shape and contrast level (Fig. 2c, also Supplementary Fig. 1). To properly distinguish and thus reconstruct the magnetization, a correlation with XMCD contrast simulation is required.

According to the experimental tube geometry, we model three different periodic magnetic domain patterns with an easy axis curling around the symmetry axis of the tube, namely azimuthal and helical magnetization with and without domain walls (Fig. 3b, also Supplementary Figs 2–4). The rolled-up nanomembrane is approximated as a closed hollow tube with one winding because of tightly rolling up along the edge and magnetostatic interwinding coupling^43^,^55^. Domains with red and blue shading refer to a clockwise and counterclockwise circulating magnetization, respectively. The helical textures resemble elliptical helices with various pitch sizes (Supplementary Fig. 3). The projected contrast patterns of helical and azimuthal magnetization with smallest possible pitch and a magnetic domain width of 1.0 μm are shown for various projection angles in Supplementary Figs 3 and 4c, respectively. The chiral property of the helix (with smallest pitch) causes the same contrast on both sides of the tube, while an azimuthal magnetization generates a reversed contrast.

The simulated contrast of an azimuthal periodically alternating domain configuration is shown in Fig. 3c and agrees reasonably well with the experimental data. For better visualization, solid and dashed lines serve as a guide to the eye. The magnetic domain configuration matching best with experiment is considered to be the true configuration. Distinct features for different projection angles are visible: At *α*=0°, the overlap is the largest leading to many small separated domains with the weakest signal due to the smallest scalar product between *M* and *k*. With increasing angles, both contrast and domain size increase. At *α*=110°, the X-ray propagation direction is almost perpendicular to the axis of the tilted tube resulting in stripe domains. The apparently bent blue domain in the experimental data can be explained as the blue contrast on each side of the tube originates from two adjacent domains that are connected by a bluish elliptical overlap. These stripes transform into domains located at the edge of the tube when reaching projection angles close to 150°. At *α*=180°, the contrast in the centre of the tube returns while preserving the edge domains. On the basis of the appearance of distinct features in the 2D projections and their evolution as a function of the projection angle, we conclude the presence of the 3D azimuthal magnetization pattern within the hollow tube. Note that we assumed a periodic arrangement of domains for simplicity that allowed to successfully discriminate between azimuthal and helical magnetization configurations, but cannot describe the small details present in the experimental XMCD contrast patterns.

The sensitivity of the 2D projection on projection angle and domain size is shown for *w*=1.7 μm (Fig. 3d) and *α*=110° (Fig. 3e), respectively. Because of tilt and eccentricity of the object, regions of the projected images with vanishing and existing XMCD contrast exhibit an oscillatory behaviour that increases with decreasing width of the magnetic domains and approaching the central region of the projected pattern (Fig. 3d). This angle dependence demonstrates the fundamental importance of acquiring projections at various angles to determine the spatial orientation of the magnetic architecture and the magnetic domains. The experimental data of clockwise (blue) and counterclockwise (red) circulating domains at *α*=110° is overlaid in Fig. 3e to determine the widths of the magnetic domains. The determined domain configuration is an alternation of (0.9±0.2) μm and (1.2±0.2) μm wide azimuthal domains. This corresponds to 11% and 15%, respectively, of the *x* axis (inset in Fig. 2d). The corresponding remanent magnetization of the top side of the tube of 0.4 times the saturation magnetization coincides with that measured by magneto-optical Kerr effect magnetometry (Fig. 2e). Note that for those simple magnetic domain patterns with straight domain walls, a correlation-based analysis of a set of 2D projections is sufficient to retrieve 3D information even if the object is tilted with respect to the rotation axis.

### Radial magnetization patterns

While T-XPEEM is a powerful tool for analysing in-plane magnetization textures, it is beneficial to use MTXM when dealing with out-of-plane magnetized architectures appearing for instance in Co/Pt and Co/Pd multilayer stacks^57^ rolled up into radially magnetized tubular structures^34^. Using MXT offers the possibility to record projections orthogonally to the tube axis and thus minimizes the aforementioned complexity due to illumination conditions in T-XPEEM. The random domain wall orientation prohibits an identification based on correlation and calls for proper reconstruction algorithms. We applied MXT to retrieve the domain pattern in radially magnetized 3D rolled-up Co/Pd multilayer stacks with a diameter of Ø≈2 *μ*m and windings separated by a non-magnetic spacer (Fig. 4a, see also Methods). Tube regions with one and multiple windings were prepared by rolling up non-rectangular nanomembranes, which allows to simultaneously investigate homogeneously magnetized (one winding) and multidomain (multiple windings) states at remanence, due to magnetostatic interaction between adjacent windings.

Selected remanent states after initial saturation (*H*≈200 kA m^−1^) along 180° are shown in Fig. 4b–d. Two sets of drift-corrected 2D projections taken at angles from 0° to 180° with step size *δ*=4° are assembled into a 3D pattern of the tube and presented in Supplementary Movies 1 and 2. The projected patterns reveal strong contrast in the centre with three different contrast levels referring to domains at front and back side of the tube with magnetization vectors pointing in- or outside the tube (Fig. 4b–d). The corresponding ambiguity of states prohibits the use of conventional microscopy of a single 2D projection. Furthermore, we emphasize that simple stacking of 2D projections will not suffice to discriminate between magnetic domain pattern on the front and back side of the tube. An identification of the local magnetization at front and back side of the tube can exclusively be given by a proper analysis of the magnetic contrast evolution with varying projection angle.

### Reconstruction algorithm

The reason for using several tilt axes in magnetic tomography in contrast to scalar tomography is the vector property of the magnetization leading to three generally independent components. While the approach described by Lade *et al.*^21^ requires three sets of projections taken at three orthogonal tilt axes, Phatak *et al.*^22^ proposed a reconstruction algorithm of the magnetic induction B from two sets exploiting the Maxwell equation ∇*B*=0. However, these procedures cannot straightforwardly be applied to XMCD-based tomography because no phase shift due to interaction with magnetic fluxes is recorded.

Our approach relies on solving systems of equations similar to those used by Manke *et al.*^8^ In this way, we analyse the contrast change with varying tilt angle that mathematically describes the derivative of the magnetization with respect to the rotation angle. Keeping in mind that XMCD is sensitive to the magnetization component pointing along the X-ray trajectory, the contribution of the magnetization component depending on the rotation axes can be determined. Using tubular architectures with well-defined magnetization orientations, for example, radial or in-plane, provides means to consider one-dimensional or 2D vector fields defined on the surface of the cylinder. In this respect, the determined magnetization component refers directly to the magnetization vector. Thus, data acquisition around one tilt axis coinciding with the symmetry axis of the tube is sufficient.

For contributions from front and back side described by *f*(*x*) and *b*(*x*) (both∝Mk), respectively, the overall XMCD contrast at projection angle *α*_*n*_ and image position perpendicularly to the rotation/tube axis *x* reads 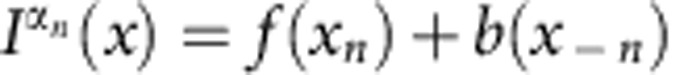 with *α*_*n*_=*α*_0_+*nδ*, 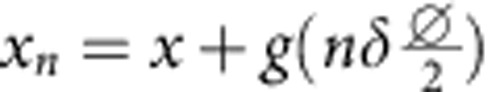 and the rotation step size *δ*. The matrix transformation due to the curvature is taken into account by 
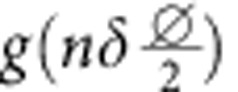
 with a lateral displacement at the very center of 
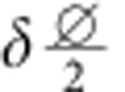
 perpendicularly to the tube axis. Thus, the XMCD contrast can be disentangled by integrating the difference between projections taken at *α*_0_ and *α*_1_:


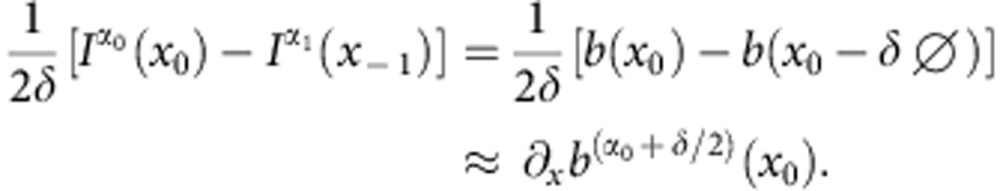


In this notation, 
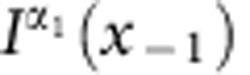
 is the XMCD contrast at *α*+*δ* shifted by 
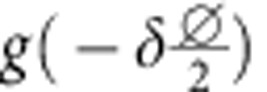
 perpendicularly to the tube axis to eliminate contributions from the front side. In case of a radially magnetized tube, the accordingly identified magnetization texture is unambiguously defined assuming a constant saturation magnetization. Spin textures with unknown magnetization orientation, such as soft-magnetic materials, require to record projections around another rotation axis. To capture the magnetic domains in a correct way, the lateral shift 
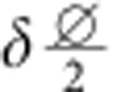
 between two subsequent projections must not exceed the domain feature size. For instance, data recorded with rotation step sizes *δ*≈4° allows to reconstruct features as small as 40 nm at the surface with curvature radii of 1 μm (1 μm away from the rotation axis). The step size has to be adjusted for each object accordingly to its dimensions and the required resolution. In our case, the smallest domain size is 75 nm (see below), which can be captured readily using *δ*=4°.

In contrast to manual tracking based on the analysis of multiple projections, the numerical approach requires only two projections when dealing with two surfaces. Drift corrections and pseudo-pattern recognition are included that already offer an assessment of the systematic error due to domain wall creeping during data acquisition and the capability to be applied to more complex samples. Considering multiple projections may further provide means to potentially discriminate contributions from more than two surfaces.

### Recontruction

**Figure 5:**
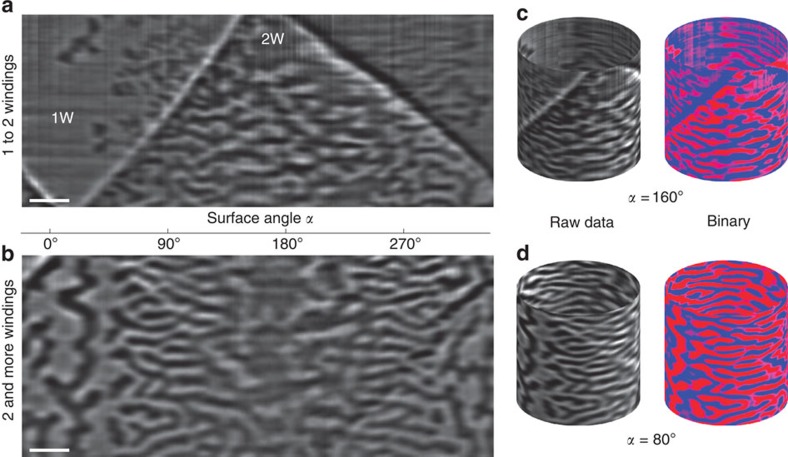
3D reconstruction of the magnetization. (**a**,**b**) Depict the unrolled magnetic domain patterns for better visualization. The transition from large isotropic to small azimuthal domains is obvious when approaching surfaces perpendicular to the initially applied magnetic field direction (0°). Dark and bright contrast refer to a magnetization pointing outside and inside the tube, respectively. Regions with one and two windings are indicated by 1 and 2 W, respectively. Scale bars, 500 nm. (**c**,**d**) 3D view of the magnetization in the tubular surfaces. In addition to the raw data (grayscale image), the processed binary data with red and blue referring to radial magnetization vectors pointing outside and inside the tube, respectively, are shown.

The reconstruction algorithm is applied to visualize the magnetization textures of radially magnetized tubular architectures in areas with one to two windings and more than two windings. Using for instance projections taken at *α*=0° and *α*=4°, we retrieve the magnetic domain patterns on front and back side of the tube with surface angles pointing along 2° and 182°, respectively (Fig. 4b). The completely reconstructed patterns are shown in Fig. 5 as unrolled magnetic domain patterns and 3D view, and in Supplementary Movies 3 and 4. Comparing the reconstructed 3D structural information (Supplementary Movie 5, also Supplementary Fig. 5) to the corresponding MXT signals allows to correlate the 3D magnetization pattern to the peculiar structure of the object (Fig. 5c,d). The processed binary data with red and blue referring to radial magnetization vectors pointing outside and inside the tube, respectively, are shown for comparison with the reconstructed magnetic domain pattern in Fig. 4e,f. We conclude that the magnetization in areas with non-overlapping windings is in a magnetic single domain state (Fig. 5a), while the area with two windings is split into multiple domains (Fig. 5a,b). We emphasize that this information cannot be obtained without performing MXT. Interestingly, we identify a multidomain state extended into the area with only one winding at the transition region between areas with one and two windings (Fig. 5a). These domains are stable during measurement, indicating that the domain walls are pinned on structural inhomogeneities as typically observed in Co/Pd multilayer stacks. Therefore, pinning dominated displacement of magnetic domain walls is expected when applying external magnetic fields, which can be described using the Kondorsky model of the magnetization reversal^44^. The appearance of the single domain state in the areas with one winding is perfectly in line with the magnetic hysteresis loop (Supplementary Fig. 6) measured on planar films before rolling up. The number of Co/Pd bilayers is optimized to ensure full remanence of the sample after magnetic saturation. The appearance of the multidomain state in the areas of the tubular object with two windings can be explained by the increased magnetostatic energy of the sample, which favours the multidomain state, when doubling the number of Co/Pd repetitions^58^. Please note that the uniaxial magnetic anisotropy of the sample is not expected to change due to the Ti spacer between each Co/Pd multilayer stack (neighbouring winding). Therefore, the reconstructed multidomain pattern in the areas with two windings shed light on the relevance of the magnetostatic coupling between the neighbouring windings of the 3D curved magnetic thin films determining its magnetic state.

## Discussion

The magnetic states in Fig. 5 are obtained after the sample has been exposed to a magnetic field applied perpendicularly to the tube axis along *α*=180° (perpendicular to the area of the sample with single winding). The morphology of the domain pattern reveals an obvious dependence on the surface angle featuring large isotropic domains with a size of more than 120 nm at the opposite side of the tube along the field direction and small domains with sizes down to 75 nm in surfaces perpendicular to the magnetic field (Figs 4e,f and 5). This difference in morphology of the domain pattern is even more pronounced in areas with two or more windings (Fig. 5b). Regions, where the magnetization is pointing perpendicular to the direction of the applied magnetic field, reveal narrow magnetic domains with lateral dimensions down to 75 nm along the tube axis and an elongation along the circumference of the tube (referred to as azimuthal domains in Fig. 4c). The appearance of this peculiar domain pattern can be understood by considering the angular dependence of the switching field of the curved surfaces: the switching field for the Co/Pd multilayers is about 60 kA m^−1^ when the field is applied along the easy axis (*α*=0°). Therefore, the maximum field of 200 kA m^−1^ available at the set-up is sufficient to saturate the sample in the angle range *α*=(−73 to 73)° taking into account the Kondorsky magnetization reversal process (Supplementary Fig. 7). There will be always regions of the sample, which cannot be saturated since the required field diverges at *α*=90°. In these areas, the multidomain pattern will be preserved. The observation of narrow azimuthal domains suggests that the applied magnetic field causes a shift of the domain walls in the areas perpendicular to the field direction, which is possible if the domain walls are of Bloch type. Hence, by reconstructing the magnetic domain pattern in the tubular object, we can gain information about the structure of the magnetic domain walls in the sample. Simultaneously, such an arrangement minimizes stray field contributions independently of the lateral expansion. These narrow azimuthal domains propagate around the tube circumference and merge into larger isotropic domains observed at *α*≈0° or *α*≈180° (Fig. 5). A similar mechanism is known for in-plane magnetized thin films with spatially varying anisotropy that favors small narrow domains in regions with large switching fields that evolves into larger domains in areas with smaller switching fields^59^.

**Figure 6:**
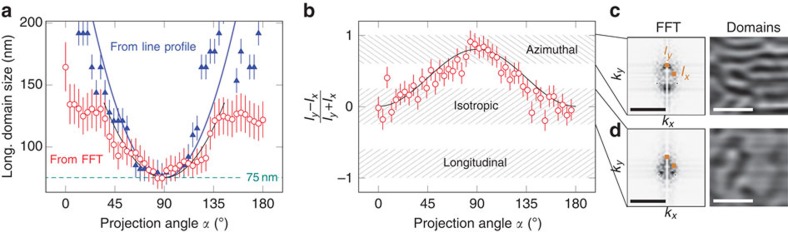
Angle dependence of the magnetic domain pattern. (**a**) Domain width in longitudinal direction reveals smallest values at *α*≈90°. Data are extracted from line profiles of real-space images (solid triangles) and from peak positions of FFT images (open circles), respectively. The uncertainties are determined by s.d. and the resolution in the reciprocal space. (**b**) Continuous transition from an isotropic/random domain configuration into an azimuthal ordering obtained from FFT. *I*_*x,y*_ is defined in **c**. Solid lines serve as a guide to the eye. Reciprocal (FFT) and real-space (domain) images of azimuthal (**c**) and isotropic (**d**) domain patterns. Dark and bright contrast refer to a magnetization pointing outside and inside the tube, respectively. Scale bars are 10 nm^−1^ and 500 nm, respectively

Analysing both morphology (isotropic or azimuthal domains) and periodicity of the domain patterns (Fig. 5) in reciprocal space reveals quantitative information about the domain patterns (Fig. 6). We perform 2D fast Fourier transformation (2D FFT) to a selected area (1 × 1 μm^2^) of the magnetic domain patterns recorded at different projection angles (Fig. 6a). This way, we assess the domain size along the tube axis to be angular dependent, which is in line with the discussion above. The largest domain size of 130 nm is observed at *α*≈0° and *α*≈180°, which decreases down to (75±9) nm at *α*≈90°. The 2D FFT data are in good agreement with the domain size extracted from the line profiles along the tube axis (compare triangle and circle symbols in Fig. 6a). The uncertainty of real-space and reciprocal-space data is determined by the s.d. and the resolution in the reciprocal space, respectively. Analysing the normalized intensity differences of the 2D FFT data, 
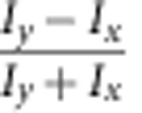
 with *I*_*x*_ and *I*_*y*_ extracted from a (1.4 × 1.4) nm^−2^ area (brown rectangles in the insets of Fig. 6b) at the location of maximum peak intensity (Fig. 6c), provides access to the spatial asymmetry of the domain pattern and allows quantification of its morphology (Fig. 6b). Projections at angles close to *α*=0° and *α*=180° do not reveal an asymmetry between the FFT intensity along vertical and horizontal directions, which is in line with the observed isotropic domain pattern. Approaching projection angles *α*≈90° results in an increase of the asymmetry to positive values indicating a transition to narrow azimuthal domains. The corresponding FFT and real-space domain patterns are shown in Fig. 6c. It is important to emphasize that we do not observe negative asymmetries suggesting the absence of domains aligned along the tube axis. In this respect, investigation of the MXT reconstruction of the 3D magnetic pattern provides access to the quantitative features of the magnetic domain pattern in tubular object with radial magnetization.

In conclusion, we presented the concept of MXT and demonstrated its potential by application to reconstruct magnetic domain patterns in 3D curved tubular surfaces with full-field X-ray microscopy utilizing XMCD as magnetic contrast mechanism. While the reconstruction with conventional scalar tomography algorithm fails, MXT revealed properly the 3D magnetization texture. Circulating magnetization patterns were identified by a correlation with XMCD contrast simulations of azimuthal and helical magnetization configurations. The absence of a preferential domain wall orientation and the corresponding ambiguity of states in more complex systems like radially magnetized Co/Pd tubes required retrieving the magnetization directly from the XMCD contrast. Analysing the evolution of magnetic domains as a function of the projection angle enabled disentangling contributions from either surface and reconstructing the magnetization. This way, the remanent states in radially magnetized tubular architectures were visualized providing means to quantify magnetic domain patterns. Depending on the magnetic field direction with respect to the surface angle and the effective out-of-plane magnetic anisotropy, different kinds of magnetic domain patterns with distinct morphology and feature sizes down to 75 nm were observed.

The proposed concept represents a platform to visualize magnetization textures in 3D curved surfaces utilizing XMCD that can be extended to multiple surfaces with virtually any shape by implementing further reconstruction algorithms. For instance, setting up and solving a linear system of equations based on multiple projections may be applied to reconstruct magnetic domains at multiple surfaces. Our method allows to study samples with thicknesses ranging from few nanometres to 200 nm due to a relatively strong absorption of soft X-rays in metals. In this respect, MXT closes the gap between investigating 3D magnetic nanostructures and bulky crystals using electron holography and neutron tomography, respectively. We envision that our technique will strongly contribute to the understanding of fundamental mechanisms and optimizing the performance of 3D tubular structures for magnetoencephalography, characterizing 3D-shaped mesoscopic objects with nanometre spatial resolution as well as on studying chirality-dependent domain wall motion enabling Cherenkov-like effects for magnons^28^, which are predicted to occur in 3D tubular architectures.

## Methods

### Sample preparation

The nickel films are evaporated via electron beam vapour deposition at an oblique angle of 60° with respect to the surface normal onto a lithographically prepatterned sacrificial photoresist layer. The internal compressive strain gradient is tuned by depositing the first and last 10 nm at a rate of 0.4 Å s^−1^ and 1.5 Å s^−1^, respectively. Dissolving the sacrificial layer in acetone releases the strained nickel film from the substrate rolling up into a tubular architecture^49^,^50^. A critical point dryer prevents the collapsing of the tube. Within a cross-beam workstation, the nickel tube is picked up and lifted via a micromanipulator and fixed onto a Pt-coated Si wafer by ion beam activated Pt deposition.

The out-of-plane magnetized [Co(0.4 nm)/Pd(0.7 nm)]_5_ multilayer stack is sputter-deposited onto a strained Ti layer and capped by 2 nm Pd. On selective release of the sacrificial layer, the heterostructure rolls up into a tubular architecture with a diameter of 2 μm and approximately three windings. While one single winding is saturated at remanence, the enlarged magnetization saturation of multiple tightly wound layers induce a multidomain state. The tube is picked up via glass capillary and micromanipulator and positioned vertically onto a rotation stage at ambient conditions.

### Magnetic imaging

The nickel sample is mounted inside an X-ray photoemission electron microscope (XPEEM, beamline UE49-PGM1 at BESSY II)^60^ onto a rotation stage. The X-ray beam hits the sample at an angle of 74° with respect to the surface normal. When resonantly exciting the valence electrons with circularly polarized light at an energy equal to the Ni *L*_3_ absorption edge, the 3D magnetization of the tube is projected onto the planar substrate. The long focal point of the X-ray beam of 32 cm provides equal foci for front and back side of the tube and ensures a parallel projection of the magnetization texture. A focus scan cannot be realized using T-XPEEM. Projections recorded at various rotation angles are used to correlate it with simulations to retrieve the actual magnetization texture within the tube.

The glass capillary with the Co/Pd tube on top is mounted onto a rotation stage between the zone plates of a magnetic transmission X-ray microscope (MTXM, beamline 6.1.2 at ALS)^61^. As the depth of focus is ∼1 μm, a higher spatial resolution is guaranteed allowing for visualizing magnetic domains down to 50 nm.

The lateral magnetic resolution of the used XPEEM and MTXM devices is about 20 nm (refs 62, 63).

### XMCD contrast simulations

The simulations of the XMCD-based 2D projections are carried out using the POV-Ray (Persistence of Vision Raytracer) framework^64^. The rolled-up nanomembrane is approximated as a closed hollow tube with one winding because of tightly rolling up along the edge and magnetostatic interwinding coupling^43^,^55^. A parallel light source resembling the propagating X-ray beam projects the magnetic spin texture onto the planar substrate. To each magnetization inside the tubular architecture an effective absorption coefficient is assigned via 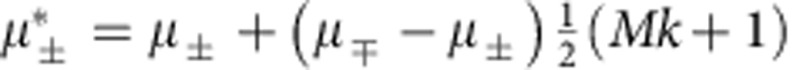 that further determines the absorbed intensity by 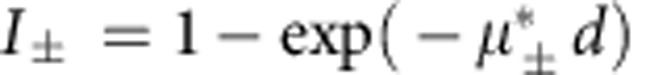. The XMCD contrast is defined as 
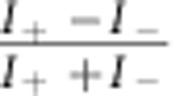
. Here *M* and *k* are magnetization and X-ray beam propagation direction, respectively, both depending on the projection angle; *μ*_±_ represent the polarization-dependent absorption coefficients at the *L*_3_ absorption edge of Ni magnetized (anti-)parallel to the beam; and *d* is the penetration depth. Spatial orientation of the tube and magnetization is considered by applying various rotation matrices. Projections at different angles are taken into account by altering the X-ray propagation direction *k*. Although the present approach performs only one iteration by assuming a homogeneous thickness of the tube to ensure time efficiency, architectures with local thickness variations may be simulated by conducting multiple iterations with smaller discretization. Moreover, as we focus on the angle- and magnetization-dependence of the XMCD patterns, relative contrast values are used.

## Additional information

**How to cite this article:** Streubel, R. *et al.* Retrieving spin textures on curved magnetic thin films with full-field soft X-ray microscopies. *Nat. Commun.* 6:7612 doi: 10.1038/ncomms8612 (2015).

## Supplementary Material

Supplementary Information Supplementary Figures 1-7

Supplementary Movie 1 The set of drift-corrected 2D projections taken at angles from 0 deg to 180 deg with 4 deg step size is assembled into a 3D pattern of the tube. The tube contains 1 winding at the back and 2 windings at the front side (0 deg projection).

Supplementary Movie 2 The set of drift-corrected 2D projections taken at angles from 0 deg to 180 deg with 4 deg step size is assembled into a 3D pattern of the tube. The tube contains multiple

Supplementary Movie 3 MXT reconstruction of the magnetic pattern of the tube containing 1 to 2 windings. Please compare with the data presented in video 1 resembling the stack of 2D XMCD projections without the reconstruction.

Supplementary Movie 4 MXT reconstruction of the magnetic pattern of the tube containing multiple windings. Please compare with the data presented in video 2 resembling the stack of 2D XMCD projections without the reconstruction.

Supplementary Movie 5 Conventional tomographic reconstruction of the rolled-up tubular architecture. The image contains the structural information only.
