# Supplementary material for: Retrieving spin textures on curved magnetic thin films with full-field soft X-ray microscopies
Source: Nat Commun. 2015 Jul 3;6:7612. doi: 10.1038/ncomms8612 (PMC4506513; doi:10.1038/ncomms8612)
Supplement: Supplementary Information — Supplementary Figures 1-7 [file ncomms8612-s1.pdf]

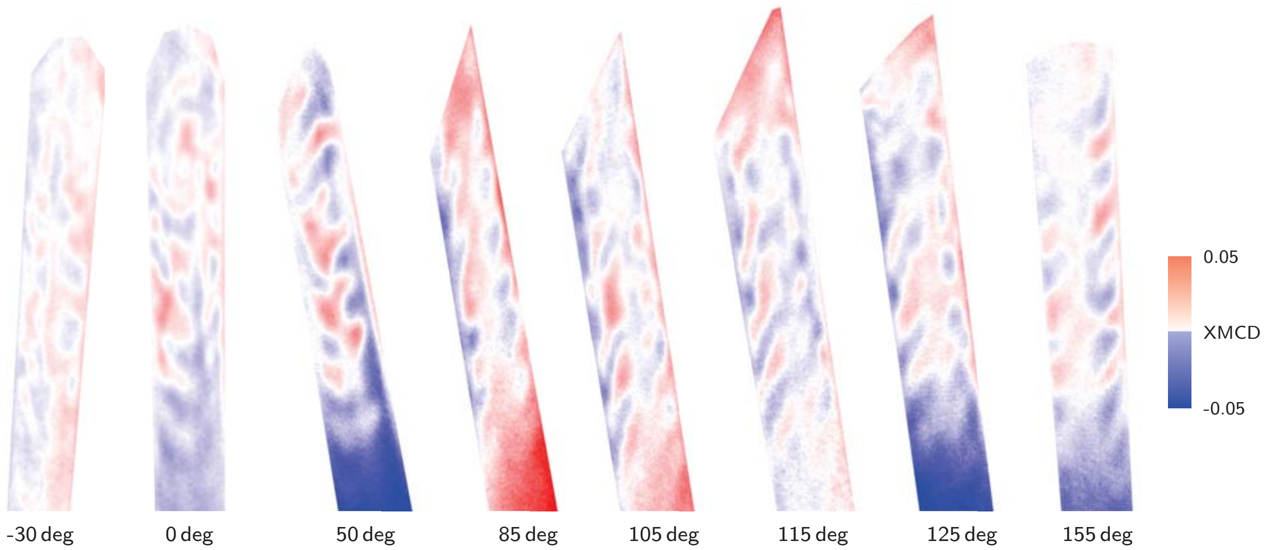

Supplementary Figure 1. Experimental 2D projections of circulating magnetization patterns in rolled-up nanomembranes utilizing XMCD in T-XPEEM. As the x-ray beam penetrates the tube at 74° with respect to the substrate, the slightly misaligned standing tube causes distinct features at various projection angles. The pronounced background of the non-magnetic substrate outside the shadow is due to beam drift during data acquisition. Considering preferentially straight domain walls in circulating magnetization textures, the magnetization texture can be reconstructed via correlating with the simulated XMCD contrast.

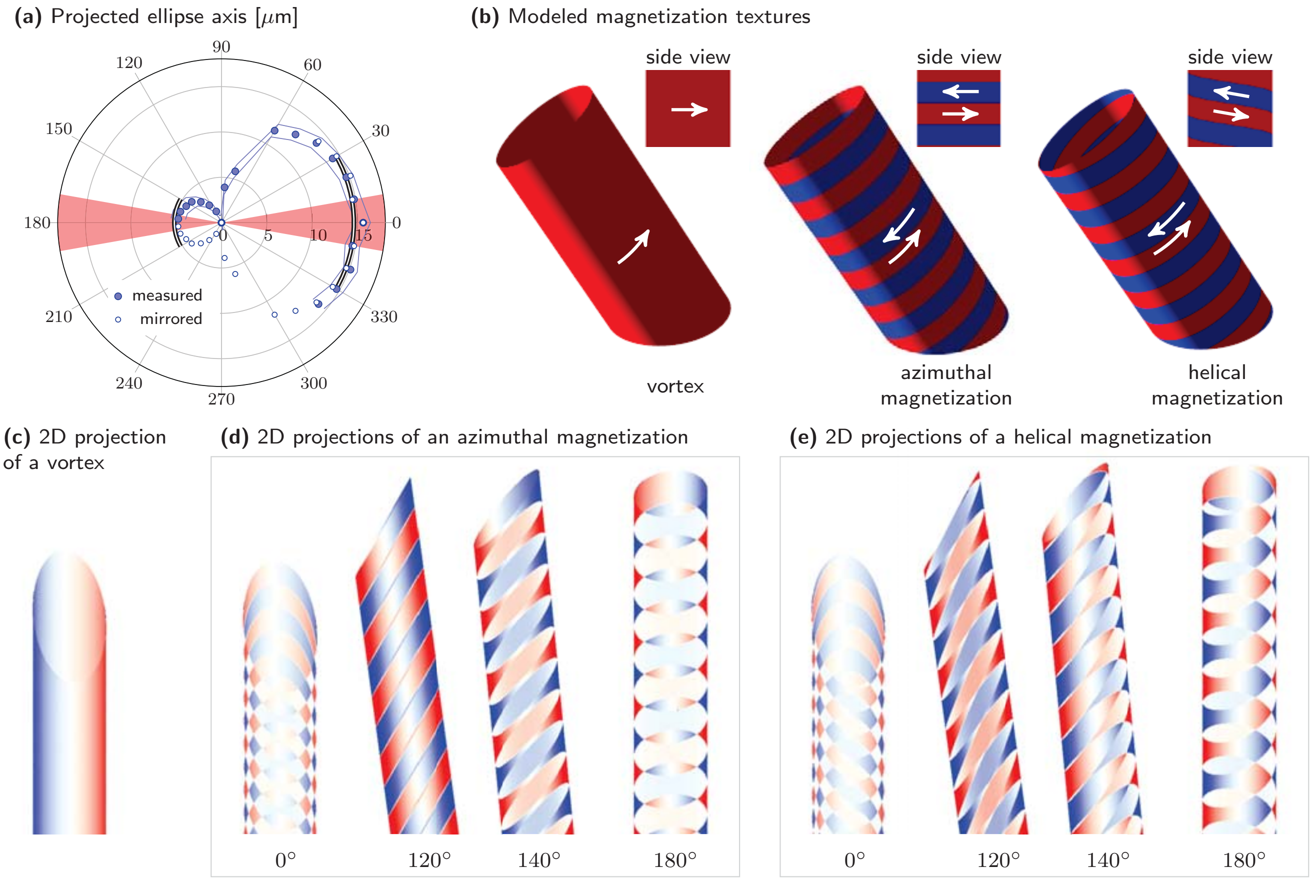

Supplementary Figure 2. Modeling the 2D projections of magnetic patterns to discriminate magnetization textures. (a) Angle dependence of the projected ellipse axis along the beam when illuminating at 74° in XPEEM to precisely determine the tube orientation. For zero and 180°, the tube is tilted towards and away from the beam, respectively. The double lines indicate the analytically calculated values. (b) Possible magnetization textures within the rolled-up nickel nanomembrane approximated as hollow tube. In addition to the 3D perspective view, the side views are shown as insets. Tube geometry is taken from experiment; Domain width is $1\,\mu$m. Panels (c), (d) and (e) depict the simulated 2D projections of vortex, azimuthal and helical magnetization for various projection angles, respectively. The 2D projection of the vortex texture is angle-independent. Whereas magnetic patterns in (d) and (e) appear similar for angles close to 0°, a distinct difference between both textures occurs for angles larger than 90°.

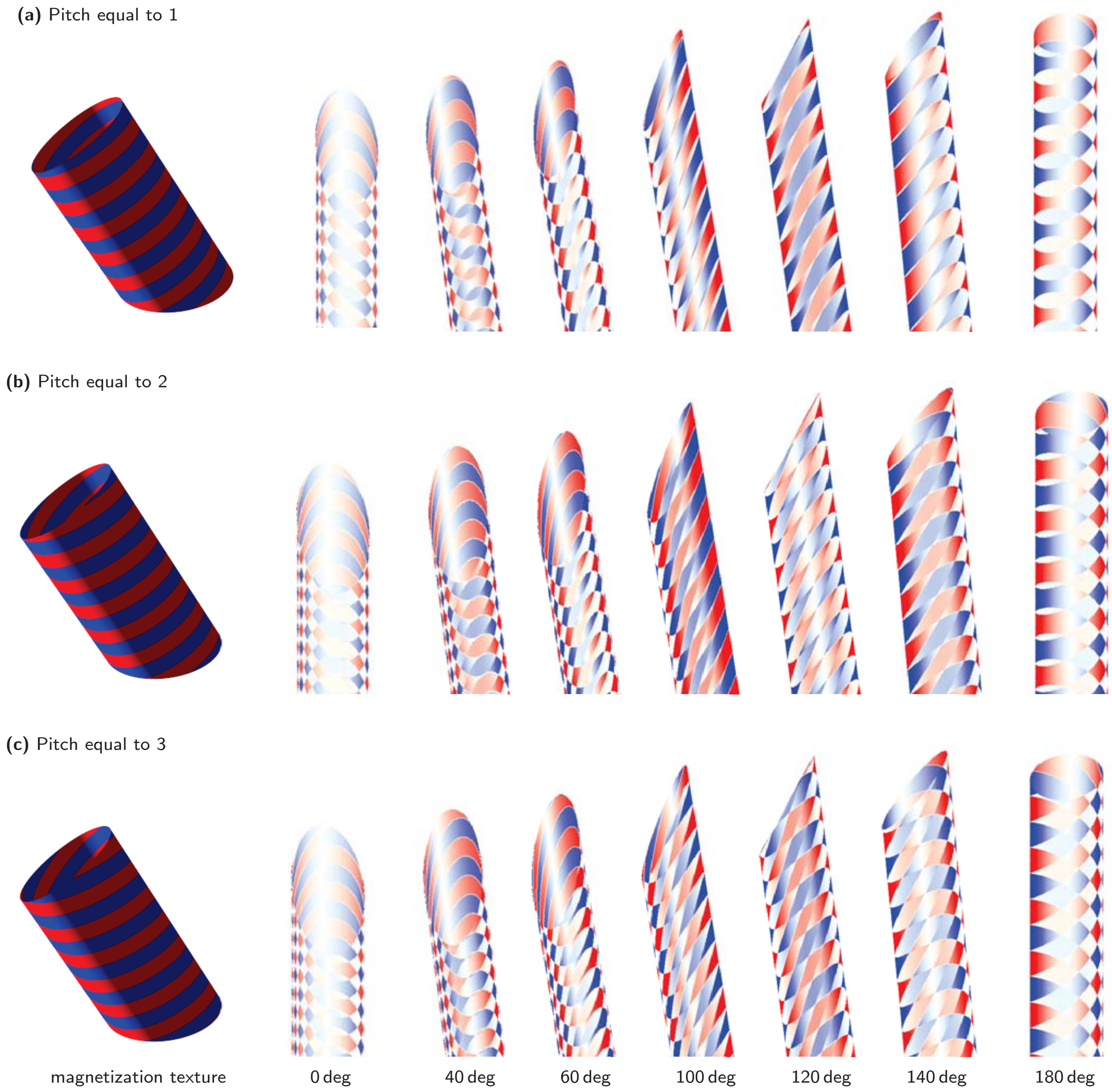

Supplementary Figure 3. Simulated 2D projections of helical magnetization textures with various pitch sizes in units of domain width. Magnetic domain width is kept constant to 0.1 times the major axis of the elliptical cross-section of the standing tube. The x-ray beam penetrates the standing tube at 74° with respect to the substrate surface and projects the 3D magnetization texture onto the planar substrate. The projected XMCD-based patterns are shown for various magnetization configurations (red and blue referring to clockwise and counterclockwise circulating magnetization) and projection angles. Both sort and sequential appearance of distinct features depend on the particular magnetization texture. The larger the pitch, the smaller the emerging patterns become.

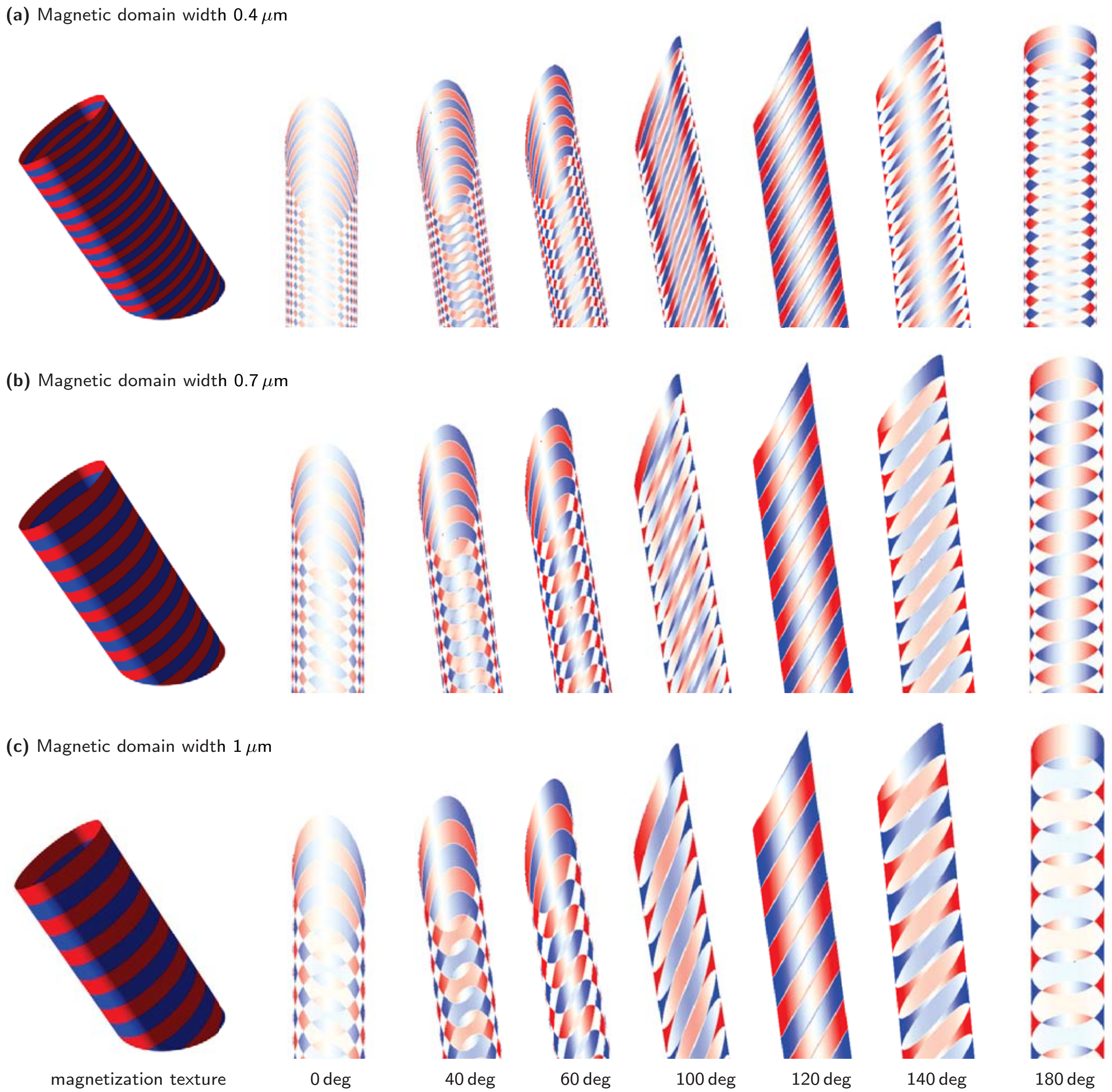

Supplementary Figure 4. Simulated 2D projections of azimuthal magnetization textures. Magnetic domain width is varied from 5% to 12.5% the major axis of the elliptical cross-section of the standing tube. The x-ray beam penetrates the standing tube at 74° with respect to the substrate surface and projects the 3D magnetization texture onto the planar substrate. The projected XMCD-based patterns are shown for various magnetization configurations (red and blue referring to clockwise and counterclockwise circulating magnetization) and projection angles. Both sort and sequential appearance of distinct features depend on the particular magnetization texture.

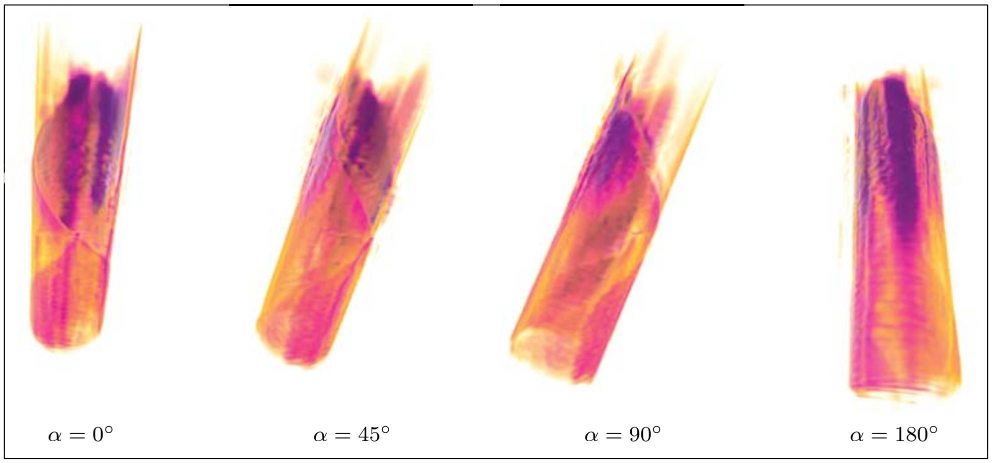

Supplementary Figure 5. 3D spatial distribution of the rolled-up nanomembrane obtained by performing conventional tomography. Snapshots at various angles reveal areas with one, two and multiple windings. Despite of these variations, the objects possess a uniaxial tubular symmetry. A complete reconstruction is presented in Supplementary Movie 5.

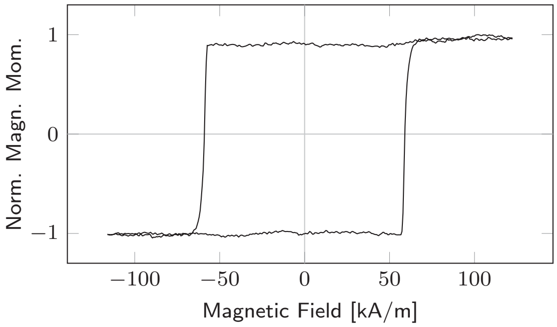

Supplementary Figure 6. Out-of-plane magnetic hysteresis loop of the $[Co(0.4\,nm)/Pd(0.7\,nm)]_5$ stack measured with polar Kerr magnetometry reveals. The sample exhibits a saturated remanent state.

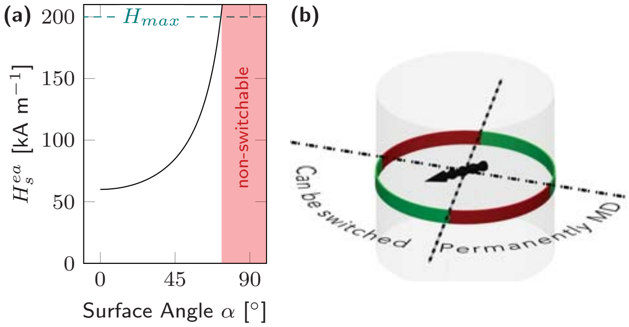

Supplementary Figure 7. Angle dependence of the magnetic switching field according to the Kondorsky mechanism. (a) A maximum field of $H_{max} \approx 200\,\text{kA m}^{-1}$ applied along 0° switches the Co/Pd magnetization in the regions with a surface angle smaller than 73° (green). The magnetization in the remaining areas (red) may only be tilted or displaced. The field direction 0° is indicated by the arrow.
